# The long-term survival characteristics of a cohort of colorectal cancer patients and baseline variables associated with survival outcomes with or without time-varying effects

**DOI:** 10.1186/s12916-019-1379-5

**Published:** 2019-07-29

**Authors:** Yajun Yu, Megan Carey, William Pollett, Jane Green, Elizabeth Dicks, Patrick Parfrey, Yildiz E. Yilmaz, Sevtap Savas

**Affiliations:** 1 Discipline of Genetics, Faculty of Medicine, Memorial University, 300 Prince Philip Drive, New Medical Education Building, St. John’s, NL A1B 3V6 Canada; 2 Discipline of Surgery, Faculty of Medicine, Memorial University, St. John’s, NL Canada; 3 Discipline of Medicine, Faculty of Medicine, Memorial University, St. John’s, NL Canada; 4 Department of Mathematics and Statistics, Faculty of Science, Memorial University, St. John’s, NL Canada; 5 Discipline of Oncology, Faculty of Medicine, Memorial University, St. John’s, NL Canada

**Keywords:** BRAF Val600Glu mutation, Colorectal cancer, Cox model with time-varying effects, Early outcome markers, Late outcome markers, Long-term follow-up, MSI, Prognostic markers, Time-varying effects

## Abstract

**Background:**

Colorectal cancer is the third most common cancer in the world. In this study, we assessed the long-term survival characteristics and prognostic associations and potential time-varying effects of clinico-demographic variables and two molecular markers (microsatellite instability (MSI) and BRAF Val600Glu mutation) in a population-based patient cohort followed up to ~ 19 years.

**Methods:**

The patient cohort included 738 incident cases diagnosed between 1999 and 2003. Cox models were used to analyze the association between the variables and a set of survival outcome measures (overall survival (OS), disease-specific survival (DSS), recurrence-free survival (RFS), metastasis-free survival (MFS), recurrence/metastasis-free survival (RMFS), and event-free survival (EFS)). Cox proportional hazard (PH) assumption was tested for all variables, and Cox models with time-varying effects were used if any departure from the PH assumption was detected.

**Results:**

During the follow-up, ~ 61% patients died from any cause, ~ 26% died from colorectal cancer, and ~ 10% and ~ 20% experienced recurrences and distant metastases, respectively. Stage IV disease and post-diagnostic recurrence or metastasis were strongly linked to risk of death from colorectal cancer. If a patient had survived the first 6 years without any disease-related event (i.e., recurrence, metastasis, or death from colorectal cancer), their risks became very minimal after this time period. Distinct sets of markers were associated with different outcome measures. In some cases, the effects by variables were constant throughout the follow-up. For example, MSI-high tumor phenotype and older age at diagnosis predicted longer MFS times consistently over the follow-up. However, in some other cases, the effects of the variables varied with time. For example, adjuvant radiotherapy treatment was associated with increased risk of metastasis in patients who received this treatment after 5.5 years post-diagnosis, but not before that.

**Conclusions:**

This study describes the long-term survival characteristics of a prospective cohort of colorectal cancer patients, relationships between baseline variables and a detailed set of patient outcomes over a long time, and time-varying effects of a group of variables. The results presented advance our understanding of the long-term prognostic characteristics in colorectal cancer and are expected to inspire future studies and clinical care strategies.

**Electronic supplementary material:**

The online version of this article (10.1186/s12916-019-1379-5) contains supplementary material, which is available to authorized users.

## Background

Colorectal cancer is an important disease to control. It is one of the most commonly diagnosed cancers in the world, causing ~ 700,000 deaths each year [1]. Many patients with colorectal cancer also experience clinically important events, such as recurrences or metastases after diagnosis. Assessing the characteristics of potential disease outcomes and identifying their predictors are critical for effective patient surveillance, and to treat and control this disease in both the short term and long term. Studies have reported that the majority of the recurrences, metastases, and deaths from colorectal cancer occur within the first few years following the diagnosis or surgery [2, 3]. The main clinical surveillance guidelines recommend up to 5 years of follow-up [4].

Clinical features (e.g., disease stage, tumor grade, histology, location), demographic variables (e.g., age at diagnosis, sex, and familial risk status), and tumor characteristics (e.g., the MSI tumor phenotype and somatic mutations, including BRAF Val600Glu mutation) are among the most commonly investigated variables in colorectal cancer [5–10]. Familial risk status may indicate familial clustering of the disease and is an interest for both the susceptibility and prognostic studies [10, 11]. Microsatellite instability (MSI) is a tumor phenotype that is characterized by defects in the DNA mismatch repair system that leads to genomic instability [12]. Generally, MSI-high tumors are associated with better patient survival [7]. BRAF Val600Glu mutation occurs in ~ 10% of the colorectal tumors, causes oncogenic BRAF activity, and promotes cellular transformation [9]. Literature reports also suggest a prognostic role for this BRAF mutation in colorectal cancer [13].

Many prognostic studies aim to identify the markers to help distinguish the patients with different outcome risks. Potential time-varying effects of markers on the patient outcomes, however, are not well-studied. Markers with time-varying effects are those whose effect direction (e.g., protective or detrimental) or size (i.e., magnitude) changes over the follow-up [14–18]. There are at least two important implications of assessing the time-varying effects of the markers in prognostic studies. First, such markers are important as they can distinguish the patients who are at increased risk of events only during specific time periods (e.g., in the short term [early event markers] or the long term [late event markers or markers with late effects]). Second, examining the time-varying effects of variables is not a standard or widely utilized research practice, which potentially leads to loss of information or inaccurate inference [14, 19, 20].

In colorectal cancer, a few studies examined the clinical, demographic, or molecular variables for time-varying effects using statistical methods. For example, disease location [21]; age, disease stage, time period of diagnosis, or tumor site [15, 16, 18]; regional cancer, age, and tumor location (pelvic/sigmoid colon) [22]; age (in two of our previous studies using subsets of the patients included in this study) [23, 24]; tumor site (left or right), grade, sex, and stage [25]; a set of genetic variations [23, 26]; and a somatic tumor alteration [27] were reported to have or tend to have time-varying effects on patient outcomes. Among the statistical methods that are used for the identification of time-varying effects are the mixture cure model [28], Cox-Aalen model, additive models with time-varying effects, and multiplicative models with time-varying effects (including piece-wise/change-point Cox proportional hazards [PH] regression model) [29, 30]. Cox PH regression model [31, 32] is one of the most widely used statistical models for time-to-event analyses in medical sciences [19]. This model has an assumption (the PH assumption) where the hazard ratio for any two groups of patients stratified by a variable remains constant over time. Violation of the PH assumption implies that the effect of the variable being investigated changes over time [31–33]. Hence, assessing the PH assumption in Cox models is an opportunity to identify the variables that have time-varying effects on patient outcomes.

While colorectal cancer is a common disease in the world, long-term prognostic characteristics and their predictors are not well known. In this study, we investigated the data collected from a prospective colorectal cancer patient cohort followed up to 19 years. Our specific aims were to examine (1) the long-term survival characteristics and (2) the associations as well as the potential time-varying effects of the widely investigated baseline clinico-demographic variables and select molecular markers on a comprehensive set of patient outcome measures.

## Methods

### Patient cohort, patient-related data, and inclusion criteria

This is an observational study. The patient cohort examined in this study was recruited by the Newfoundland Colorectal Cancer Registry [34, 35]. This registry includes 750 incident cases, who were diagnosed with colorectal cancer in Newfoundland and Labrador (NL) between January 1999 and December 2003. These patients constituted 64% of the eligible patients who were diagnosed within this time frame. Among the 750 patients recruited by the registry, clinical and prognostic data of 744 patients were available in the registry and were provided to the study team. Out of 744, 738 colorectal cancer patients with stage I–IV disease and age at diagnosis ≤ 75 were included in the present study (5 patients with in situ/stage 0 tumors and 1 patient with > 75 years of age were excluded). In this study, clinical, pathological, demographic, and molecular markers that are most widely examined by the colorectal cancer research community and present in at least 5% of the patient cohort were selected for assessment (Table 1). Tumor MSI and BRAF Val600Glu mutation statuses were determined previously as described in Woods et al. [35], and familial risk status was assessed as described in Green et al. [34]. Information on the clinical, pathological, and demographic as well as the vital status, cause of death, recurrence, and metastasis was collected over time using several resources as described in Negandhi et al. [36] that included patient follow-up questionnaires, medical records (e.g., physician notes/assessments, pathology, surgery, and autopsy reports/death certificates), Provincial Tumor Registry-NL/Dr. H. Bliss Murphy Cancer Centre, and NLCHI (Newfoundland and Labrador Centre for Health Informatics). The distinction between loco-regional recurrence and distant metastasis was based on the pathology reports, diagnostic imaging reports, location of tumors, or physician’s notes. If a tumor had occurred in the field of the primary resected tumor, including proximal or distal to the site of anastomosis, it was classified as recurrence. Distant recurrences were classified as metastasis based on the location and clinical assessment of the origin of the tumor and physicians’ opinions.

**Table 1:** Baseline characteristics of the patient cohort

Variable	Number	Percentage (%)
Age at diagnosis		
Median (range)	62.37 (20.70–75.01)	–
Sex		
Male	452	61.25
Female	286	38.75
Familial risk		
Low risk	355	48.10
High/intermediate risk	362	49.05
Unknown	21	2.85
Location		
Colon	507	68.70
Rectum	231	31.30
Stage		
I	113	15.31
II	245	33.20
III	227	30.76
IV	153	20.73
Histology		
Non-mucinous	646	87.54
Mucinous	92	12.47
MSI status		
MSI-L/MSS	636	86.18
MSI-H	73	9.89
Unknown	29	3.93
BRAF Val600Glu mutation status		
Wild-type	591	80.08
Mutant	80	10.84
Unknown	67	9.08
Grade		
Well/moderately differentiated	653	88.48
Poorly differentiated	73	9.89
Unknown	12	1.63
Adjuvant chemotherapy treatment		
No	387	52.44
Yes	346	46.88
Unknown	5	0.68
Adjuvant radiotherapy treatment		
No	565	76.56
Yes	151	20.46
Unknown	22	2.98

*MSI* microsatellite instability, *MSI-H* microsatellite instability-high, *MSI-L* microsatellite instability-low, *MSS* microsatellite stable

The last date of follow-up in this cohort was January 2018. An overview of the characteristics of the clinico-demographic variables and molecular markers of the patient cohort is shown in Table 1.

### Statistical analyses

#### Assessing the collinearity among the variables

We assessed and ruled out the potential correlation between the categorical variables (Table 1) based on the pair-wise Pearson’s correlation coefficient value (Additional file 1: Table S1).

#### Survival outcomes

A set of widely investigated survival outcomes were examined in order to conduct a comprehensive investigation. The endpoints of overall survival (OS), disease-specific survival (DSS), recurrence-free survival (RFS), metastasis-free survival (MFS), recurrence or metastasis-free survival (RMFS), and event-free survival (EFS) were death from any cause, death from colorectal cancer, diagnosis of local recurrence, diagnosis of distant metastasis, diagnosis of recurrence or metastasis, and diagnosis of recurrence, metastasis, or death from colorectal cancer, respectively. Survival times were calculated starting at the date of diagnosis till the date of the first occurrence/observation of the endpoint (or the date of last contact); in multi-event outcomes, the latter date was the date of the first event. In each survival outcome, data were censored at the date of last contact for patients who have not experienced the events of interest during their follow-up. Data on the survival outcomes are summarized in Table 2.

**Table 2:** Number of events in the survival outcomes examined in this study

Survival status	Number	Percentage (%)
OS status		
Alive	290	39.30
Died	448	60.70
DSS status		
Death from other causes or alive	399	54.07
Death from colorectal cancer	192	26.02
*Unknown	147	19.92
RFS status		
Recurrence (−)	661	89.57
Recurrence (+)	77	10.43
MFS status		
Metastasis (−)	587	79.54
Metastasis (+)	151	20.46
RMFS status		
Recurrence or metastasis (−)	542	73.44
Recurrence or metastasis (+)	196	26.56
EFS status		
Recurrence, metastasis, or death from colorectal cancer (−)	359	48.64
Recurrence, metastasis, or death from colorectal cancer (+)	287	37.67
*Unknown	101	13.69

*DSS* disease-specific survival, *EFS* event-free survival, *MFS* metastasis-free survival, *OS* overall survival, *RFS* recurrence-free survival, *RMFS* recurrence/metastasis-free survival

*This is because the cause of death information was missing for some patients

#### Kaplan-Meier and Cox regression analyses and proportional hazards assumption test

IBM SPSS Statistics (version 25) was used for Kaplan-Meier method that generated the survival curves. Univariate Cox models were fitted for variables for each of the survival outcomes. PH assumption test [37] was performed using cox.zph function [38] in R (ver. 3.5.0) [39] using the default “km” function to obtain the transformed survival times. Multivariate Cox models were constructed using backward selection method, and when the PH assumption was violated, Cox model with time-varying effects (assuming piece-wise constant hazard ratios—this model is also called change-point Cox model [29, 30]) was used. Full multivariate models with all baseline variables were first checked for the PH assumption. For the variables that violated the PH assumption, cutoff time points before and after which the PH assumption was satisfied were obtained, starting from the variable with the lowest *p* value of the PH assumption test. The proper cutoff time points were selected based on the approach described in Pavelitz et al. [27] and Klein and Moeschberger [29]. In this study, a set of cutoff time points ranging from 0.5 to 18.5 years and with increments of 0.5 years was considered. The proper cutoff time point is ideally the one which makes the model (1) having the largest maximized log partial likelihood and (2) with the PH assumptions being satisfied before and after the time point. If the model with the largest maximized log partial likelihood did not satisfy the proportional hazards at both time intervals separated by the tested time point, then the one with the second largest maximized log partial likelihood value was tested. This step was repeated until a model was obtained that satisfied the criteria. The corresponding cutoff time point was then deemed to be the proper cutoff time point. In cases when the cutoff time points made the model having an infinite upper limit of the 95% confidence interval (CI) for hazard ratio (HR) of a variable, the cutoff point with the next largest maximized log partial likelihood was considered. This is because infinite limits suggest that a valid effect estimation cannot be made. In addition, rarely a proper cutoff time point for a variable was not identifiable. For example, for stage III in the OS analysis, a single time point that satisfies the PH assumption in both time periods (before and after the time point) was not identified. We then introduced additional time points in one of the time periods where the PH assumption was violated. However, this step did not identify any proper time points in this region. In this case, we analyzed this variable with the next one in line (i.e., the next variable with the smallest *p* value of the PH assumption test) and tested all the possible combinations of the cutoff time points to identify the proper cutoff points of both variables at the same time. Once the proper cutoff time points were identified and included in the model, variables with *p* values ≥ 0.05 in the model were removed one by one, starting with the one with the largest *p* value. During this process, if any of the remaining variables violated the PH assumption, the cutoff time point was identified/re-identified for this variable based on the method described above, followed by re-fitting of the model. The variables in the final model for each outcome measure reported in this manuscript have a *p* value < 0.05 either over the follow-up time (i.e., variables with no time-varying effects) or in at least one time period defined by the cutoff time points (i.e., variables with time-varying effects).

Age at diagnosis was examined as a continuous variable in this study. A *p* value < 0.05 was considered significant. All statistical analyses were performed by R (ver. 3.5.0) [39] or IBM SPSS Statistics (version 25).

## Results

### Characteristics of the survival outcomes in the patient cohort

Baseline characteristics of the patients and information on the outcome measures investigated are shown in Tables 1 and 2. The median follow-up time was 9.36 years and with a range of 0.04 to 19.00 years. Among the 738 colorectal cancer patients, 448 (~ 61%) died by the end of the follow-up period. The number of deaths caused by colorectal cancer (*n* = 192) accounted for ~ 43% of all deaths. Stage IV patients had the highest rate of death (death from any cause and death from colorectal cancer were recorded for the 94.8% and 86.9% of the stage IV patients, respectively). In addition, 77 patients (~ 10%) had experienced at least one local recurrence, and 151 individuals (~ 20%) had experienced at least one metastasis. The majority of the patients diagnosed with recurrence or metastasis were stage II or III patients, whereas ~ 14% of the patients who experienced recurrences and ~ 7% of the patients who experienced metastases during their follow-up were stage I patients. Around 27% (*n* = 196) of the patients experienced either recurrence or metastasis. The proportion of patients who had experienced both recurrence and metastasis was low (*n* = 32 patients; 4%), yet almost half of the patients who had recurrence also had metastasis. Approximately 73% of the patients who were diagnosed with recurrence or metastasis died from colorectal cancer (110 out of 150 patients with complete data on recurrence, metastasis, and cause of death). Of the 448 patients died during the follow-up period, 171 patients experienced recurrence or metastasis before they passed away. Overall, ~ 38% of the patients had at least one disease-related and clinically important event (i.e., recurrence, metastasis, or death from colorectal cancer) during the follow-up.

### Survival patterns over time

**Fig. 1:**
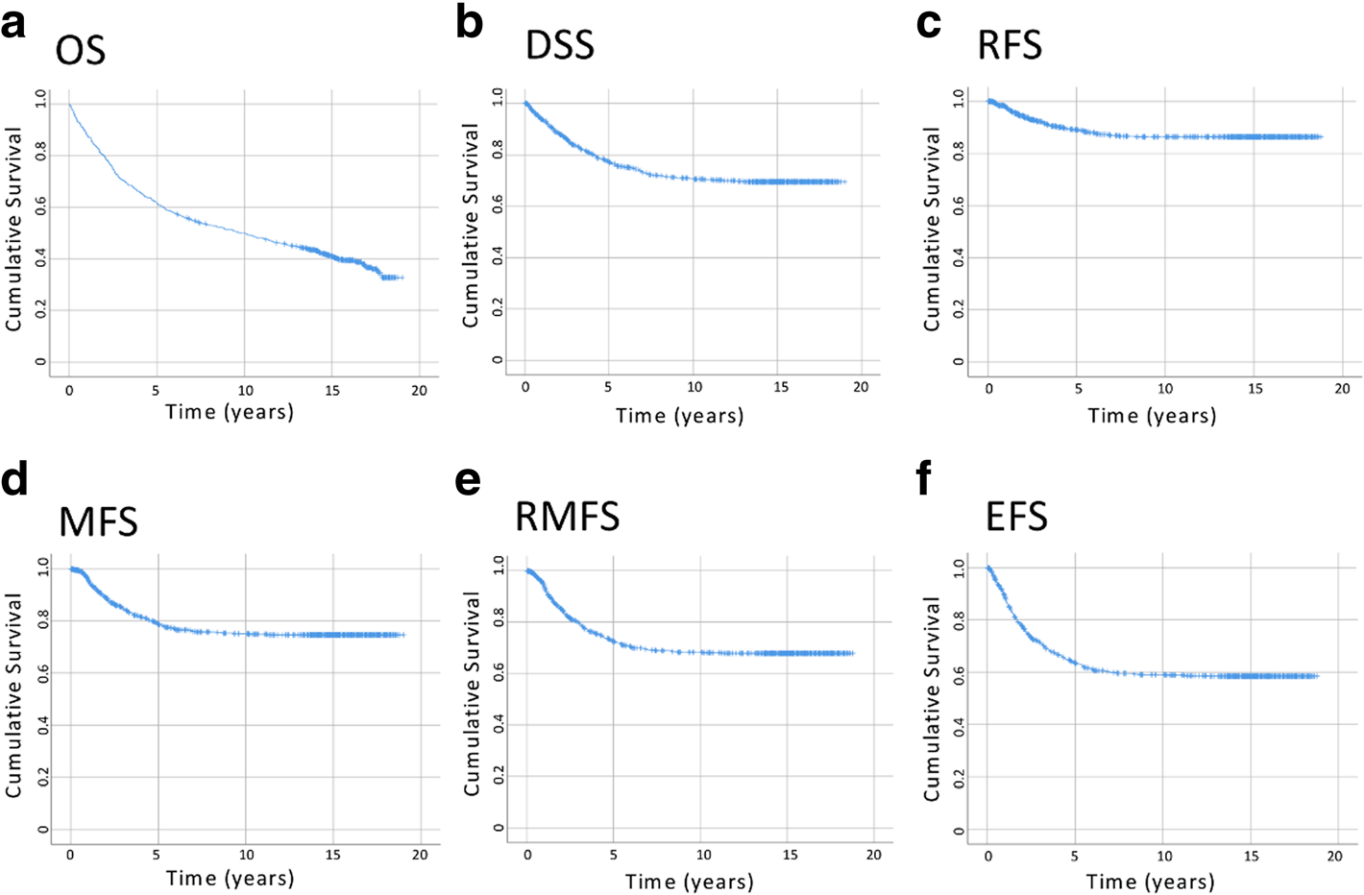
**a**-**f** Kaplan-Meier curves of the survival outcomes. DSS, disease-specific survival; EFS, event-free survival; MFS, metastasis-free survival; OS, overall survival; RFS, recurrence-free survival; RMFS, recurrence/metastasis-free survival

Kaplan-Meier curves for the outcome measures examined are shown in Fig. 1. Unlike death from all causes (OS: Fig. 1a), the majority (85%) of the deaths due to colorectal cancer occurred within 6.2 years post-diagnosis (DSS: Fig. 1b). Similarly, the majority (85%) of the first recurrences and/or metastases were diagnosed within ~ 5 years after the disease diagnosis (5.1 years for RFS; 4.9 years for MFS; ~ 4.5 years for RMFS; Fig. 1c-e). As for the EFS that considers the three most important disease-related events, 85% of the first of any of these events were observed within the first ~ 4.5 years after diagnosis (Fig. 1f). It is important to note that within this group of patients (i.e., with a positive status of recurrence, metastasis, or death from colorectal cancer), only a small portion of the patients (5%) experienced their first disease-related events after 6 years following the diagnosis of colorectal cancer.

### Variables with or without time-varying effects on survival outcomes

#### Univariate analyses

**Fig. 2:**
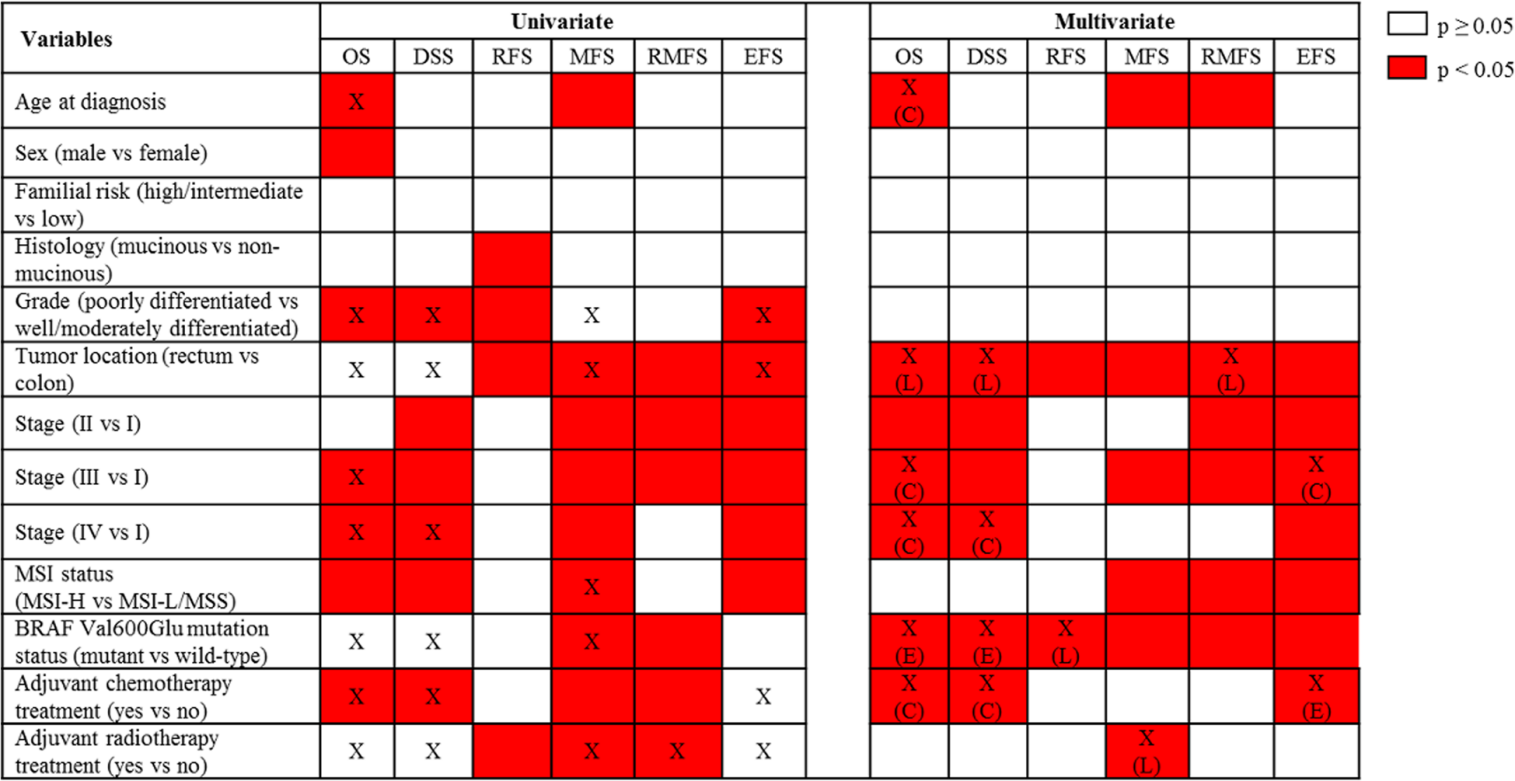
Associations between clinico-demographic/molecular markers and the survival outcomes. C, change of effect size; DSS, disease-specific survival; E, early-effect; EFS, event-free survival; L, late-effect; MFS, metastasis-free survival; MSI, microsatellite instability; MSI-H, microsatellite instability-high; MSI-L, microsatellite instability-low; MSS, microsatellite stable; OS, overall survival; RFS, recurrence-free survival; RMFS, recurrence/metastasis-free survival. X, variables that violated the PH assumption in the univariate Cox analyses or violated the PH assumption and found to have time-varying effects in the multivariate Cox analyses

Univariate associations between clinico-demographic and molecular variables and survival outcomes are summarized in Fig. 2. All variables investigated, except the familial risk status, were associated with at least one survival outcome. Several variables also violated the PH assumption of the Cox regression models. For these variables, Kaplan-Meier curves showing the survival probabilities over time are depicted in Additional file 1: Figure S1-S3. Those variables that violated the PH assumption and were significantly associated with the outcomes (univariate Cox regression *p* value < 0.05) tended to have separated curves with no visible crossing of the curves (type A variables; Additional file 1: Figure S1 and Figure S3). In contrast, it was clearly observable that those variables that violated the PH assumption but were not significantly associated with the outcomes (i.e., univariate Cox regression *p* value ≥ 0.05) tended to have their curves crossed (type B variables; Additional file 1: Figure S2).

#### Multivariable Cox regression models

Multivariable models are shown in Additional file 1: Tables S2-S7, and the main findings are summarized in Fig. 2. Age at diagnosis was associated with overall survival as well as metastasis-related outcomes (Additional file 1: Tables S2, S5-S6). The effect size of this variable on the risk of death from any cause became slightly larger after 10.5 years post-diagnosis (Additional file 1: Table S2). In contrast, increasing age at diagnosis was associated with decreased risks of MFS (HR 0.98; Additional file 1: Table S5) and RMFS (HR 0.98; Additional file 1: Table S6). Other demographic variables (sex and familial risk) as well as tumor histology and grade were not associated with any of the survival outcomes.

Tumor location and BRAF Val600Glu mutation status were associated with all outcomes. The effects of these two variables either remained constant or varied with time on different disease outcomes (Fig. 2; Additional file 1: Tables S2-S7). For example, the results of RFS, MFS, and EFS analyses showed that rectal cancer patients compared to colon cancer patients had shorter times to events throughout the follow-up time with no detectable time-varying effects (Additional file 1: Tables S4-S5, and S7). In the OS analysis, no significant difference between the rectal and colon cancer patients was detected prior to 2 years following diagnosis. However, after this time point, the risk for rectal cancer patients became significantly higher (HR 1.68; Additional file 1: Table S2). Also, while in the early years RMFS and DSS times did not significantly differ between the rectal and colon cancer patients, after 3 years in RMFS and after 6.5 years in DSS, the event risk significantly increased for the rectal cancer patients (HR 3.91 for RMFS and 5.97 for DSS; Additional file 1: Tables S3 and S6). The presence of BRAF Val600Glu mutation was associated with shorter overall and disease-specific survival times within the first 2.5 years post-diagnosis (HR 2.18 for OS and 3.05 for DSS), but not after that (Additional file 1: Tables S2-S3). This mutation was also significantly associated with an increased risk of recurrence after 4 years following diagnosis (HR 7.10; Additional file 1: Table S4). Last, patients with this tumor mutation had shorter MFS, RMFS, and EFS times without any time-varying effects (Additional file 1: Tables S5-S7).

Stage was associated with all outcomes except the risk of recurrence (Fig. 2). Patients with advanced stages had generally increased risks of outcome events, stage IV disease was a strong predictor of death, and stage III disease was a predictor of metastasis (Additional file 1: Tables S2-S3, S5-S7). For this variable, time-varying effects were found on death-related outcomes (i.e., OS, DSS, and EFS). Specifically, compared to stage I patients, stage III and stage IV patients had a much higher risk of death from any cause within the first year following diagnosis than later (Additional file 1: Table S2). Similar to this, for stage IV patients, the risk of death from colorectal cancer was much higher during the first year post-diagnosis (Additional file 1: Table S3). Additionally, the risk of having at least one disease-related event for stage III patients (EFS) was much higher within the first 1.5 years following diagnosis, which then decreased in magnitude (HR 6.02 within the first 1.5 years post-diagnosis versus 2.99 after that; Additional file 1: Table S7). The effects of disease stage on other outcome measures did not change over time.

Tumor MSI phenotype was associated with only metastasis-related outcome measures (MFS, RMFS, and EFS; Fig. 2). MSI-H tumor phenotype had a protective effect (Additional file 1: Tables S5-S7). Unlike other variables, MSI status had no time-varying effects.

Last, the two treatment-related variables, adjuvant chemotherapy and adjuvant radiotherapy treatment statuses, showed different association patterns in this observational cohort. While the associations of the adjuvant chemotherapy treatment were detected in death-related outcomes (i.e., OS, DSS, and EFS), adjuvant radiotherapy treatment was only associated with MFS (Fig. 2). These effects were non-proportional during the follow-up (i.e., varied over time). Specifically, within the first year following diagnosis, adjuvant chemotherapy had strong and significant protective effects on OS, DSS, and EFS, after which this effect was not detectable in the EFS analysis but was still significant in the OS and DSS analyses, albeit with a decreased effect size (HR 0.05 for OS, 0.15 for DSS, and 0.40 for EFS within the first year post-diagnosis and 0.56 for OS and 0.50 for DSS after this time point) (Additional file 1: Tables S2-S3 and S7). Whereas, compared to patients who did not receive adjuvant radiotherapy, patients who received adjuvant radiotherapy had an increased risk of metastasis (HR 6.00) after 5.5 years following diagnosis (Additional file 1: Table S5).

## Discussion

In this study, we examined the survival characteristics of a prospective cohort of colorectal cancer patients (*n* = 738) followed up to 19 years and the association of a set of baseline variables with outcome measures. This long follow-up time makes it an excellent resource for the investigation of prognostic characteristics in both the short and long terms. Our results show the survival characteristics in this patient cohort over a long follow-up time; describe the relationships between baseline clinical, demographic, and select tumor molecular markers and a comprehensive set of patient clinical outcomes; present interesting findings regarding variables with time-varying effects; and identify a set of candidate early-effect and late-effect markers that can help distinguish patients who are at increased outcome risks during specific time periods following diagnosis.

### Long-term survival characteristics of the patient cohort

Overall, some of our results supported the previous literature findings, and some others provided new insights. Characteristics of the patient cohort and survival probabilities are shown in Tables 1 and 2 and Fig. 1. As expected, a portion of the patients experienced disease progression (i.e., recurrence/metastasis), and this was strongly linked to death from colorectal cancer. The majority (85%) of the first recurrence and/or metastasis (Fig. 1c-e) and deaths from colorectal cancer (Fig. 1b) were clustered during the first ~ 4.5–5 and ~ 6 years, respectively. These findings, similar to other reports, emphasize the initial years after diagnosis as a critical window of time for colorectal cancer patients [2–4]. However, in some patients, the first recurrence or metastasis happened after the first 5 years (15.8% and 13.3% of the events, respectively). This raises the question of whether the medical surveillance should be extended beyond the most recommended time frame of 5 years for the patients who did not experience disease progression until then. Similar observations and suggestions were made by others [40, 41]. On the positive side, our results (Fig. 1f) also showed that when a patient survived the initial 6 years without any disease-related event (recurrence, metastasis, or death from colorectal cancer), their risk for these disease outcomes became much less afterwards (~ 95% of the patients who had any of these events had their first events or died within the first 6 years). This suggests that the long-term consequences of colorectal cancer become minimal once a patient survives the first 6 years event-free.

### Modeling time-varying effects and previous literature findings in colorectal cancer

In order to examine the relationships between the variables and outcome measures, we applied both the univariate and multivariate analyses. In these analyses, we aimed to explore the variables for their constant as well as potential time-varying effects. We note that while the term “effect” suggests a direct effect of the variable, it should not be taken literally—it rather reflects an association. In our case, variables with constant effects are those that satisfy the proportional hazards assumption of the Cox model and for which the hazard ratio estimations throughout the follow-up time remain constant. Variables with time-varying effects, on the other hand, are those that have their effects (i.e., HRs) change over time. This also means that a marker’s effect may only be detectable or obvious during a specific time period, or the direction of the marker’s effect may change over different time periods. Intuitively, to identify such variables, data obtained from cohorts followed up for a long time, like the cohort examined in this study, is needed. Previous studies reported that age, sex, grade, stage, tumor location/site, a somatic alteration, and a few genetic polymorphisms had potential time-varying effects in colorectal cancer [15, 16, 18, 22–27]. However, to our knowledge, only a few of these studies identified the time periods using the patient data, which reflect the patterns of effects on survival times [23, 26, 27], as we did in this study. Also, in our study, we used Cox model with time-varying effects assuming piece-wise constant hazard ratios, which provided simple (i.e., one time point per variable) and potentially clinically meaningful information.

### Time-varying effects identified in the univariate analyses and implications for multivariable modeling

In our study, distinct patterns of survival probability for variables with non-proportional effects (type A and type B variables) were observable after univariate analyses and assessment of the PH assumption (Additional file 1: Figure S1-S3). It should be noted that the differences between type A and type B variables have implications for researchers: characteristics of type B variables (i.e., which do not have a significant *p* value in the univariate analyses) indicate that such variables may be excluded from multivariable modeling if the researchers select the covariates based on the univariate *p* values. Such an exclusion could then lead to the omission of important variables (e.g., those with potential time-varying effects) in the final models.

### Multivariable models and associations detected with or without time-varying effects

#### Demographic factors and their relation to outcome measures

Multivariable models that considered the time-varying effects yielded a number of interesting findings (Additional file 1: Tables S2-S7). Regarding the demographic features, increasing age at diagnosis was associated with a small but significant increased risk of mortality throughout the follow-up time. This risk became slightly larger after the initial 10.5 years (OS; Additional file 1: Table S2). It is not surprising that younger patients had a lower risk of death, as they normally would have fewer comorbidities, lower chances of dying from other causes, and are likely to receive aggressive and intense treatments [42] that may contribute to their longer survival times. The slight increase in the risk of death after a decade can be explained by the aging of the patients in the cohort. On the other hand, small but long-term effects were detected for age at diagnosis on metastasis-related outcomes (MFS and RMFS) where decreased age was associated with worse MFS/RMFS times (Additional file 1: Tables S5-S6). It is reported by other studies that younger colorectal cancer patients present with advanced diseases [42, 43]. In our cohort 32.8% and 27.7% of the younger patients (age at diagnosis < 65) and older patients, respectively, were diagnosed with a stage III disease—this may explain the increased metastasis risk in the young patients. In contrast to age, another demographic variable, sex, was not associated with any of the survival outcomes examined in this study. The role of patient sex in prognosis is controversial: some studies support that female patients have a better prognosis compared to male patients [44–46] while others do not find such a sex-based difference [21, 47]. We observed a better survival for female patients in the univariate analysis, but this association was not retained in the multivariable models. Additionally, consistent with other studies [10, 48], familial risk status, while it is a risk factor for the development of colorectal cancer [11, 49], had no significant relation to any survival outcomes investigated. Therefore, in this cohort, age at diagnosis has emerged as the only demographic factor with a predictive role.

#### MSI and disease stage and their relation to outcome measures

In our analysis, MSI status was predictive of only metastasis-related outcomes (MFS, RMFS, and EFS; Additional file 1: Tables S5-S7), and its effects remained stable during the entire follow-up. MSI-H is a known marker with protective effects on patient survival [7, 50], possibly due to its biological effect on metastasis through its association with increased immune cell infiltration [51]. Thus, our results are consistent with these previous findings but additionally emphasize that the MSI status predicts the risk of metastasis even long after the diagnosis. As the most important prognostic marker, stage was a predictor of the majority of the outcome measures investigated (Fig. 2). As expected, increased disease stage was generally associated with increased risk of events, but in some cases, the hazard ratios significantly differed before and after a time point. Interestingly, such effects were detected in death-related outcomes. Specifically, fluctuating HRs were detected for stage III patients in the OS and EFS analyses and for stage IV patients in the OS and DSS analyses. In these cases, the risk of event was much higher for the patients immediately after the diagnosis (i.e., within 1–1.5 years) compared to later. This time relationship may be attributed to the advanced disease at diagnosis and/or the post-surgical complications that are known to lead to early death [52–54]. We note that in a previous study on OS, similar findings (i.e., non-proportional effects of stage III and stage IV disease) were reported [25].

#### Tumor location and its relation to outcome measures

Two variables were associated with all outcomes examined in this study, and tumor location was one of them. Tumor location is one of the most widely examined clinical variables in colorectal cancer and is used in the clinic for prognostic estimations as well as surveillance and treatment-related decisions. In our study, rectal tumors compared to colon tumors were associated with worse RFS, MFS, and EFS times throughout the follow-up with no time-varying effect (Additional file 1: Tables S4-S5, S7). It is known that rectal cancer patients have a higher risk of recurrence and metastasis [55, 56], which is also shown by our results. However, our results additionally showed that the rectal tumors had sustained these constant/continuous effects over a long time after the diagnosis. In contrast, in the OS, DSS, and RMFS analyses, we observed time-varying effects of tumor location. The DSS and RMFS model data were particularly interesting. In both models, rectal cancer patients tended to have worse outcomes compared to colon cancer patients, but this difference reached significance only after certain time points. In the RMFS model, the risk for increased recurrence/metastasis became significantly higher for rectal cancer patients only after the initial 3 years. Since recurrence and metastasis indicate disease progression, RMFS data may be particularly relevant for clinical surveillance purposes and may suggest that the rectal cancer patients who survived the first 3 years without disease progression may need to be carefully surveilled after this time period. Additionally, a similar and a later effect was observed in the DSS model, where the risk of death from colorectal cancer significantly increased for the rectal cancer patients after 6.5 years. The non-proportional effect of tumor location on DSS has been observed by others as well [21]. In our study, the increased risk of disease progression for rectal cancer patients after 3 years (Additional file 1: Table S6) may explain their increased risk of disease-specific death after 6.5 years (Additional file 1: Table S3).

#### BRAF Val600Glu mutation and its relation to outcome measures

Like tumor location, BRAF Val600Glu mutation status was associated with all outcome measures (Additional file 1: Tables S2-S7). This mutation is one of the most studied tumor mutations in colorectal cancer as well as other cancer sites, such as ovarian cancer [57], thyroid cancer [58], lung cancer [59], and melanoma [9]. In our study, patients with this tumor mutation had increased risk of two metastasis-related outcomes throughout the follow-up (the highest risk being associated with metastasis-free survival; HR 3.46). Such a relationship between mutant BRAF and metastasis was previously reported in other cohorts [60, 61]. This mutation was also associated with a shorter time to recurrence after 4 years. It is not immediately clear how this mutation may influence the recurrence risk in colorectal cancer, but the association of this mutation with tumor recurrence has been reported in papillary thyroid cancer as well [58]. In addition to these, previously, BRAF Val600Glu mutation has been associated with the increased risk of mortality in colorectal cancer [13, 61–64]. In our study, in two death-related outcomes (OS and DSS), this mutation emerged as a predictor of death early after diagnosis (within the first 2.5 years). Interestingly, this group of patients also tended to have better DSS times if they survived the initial 2.5 years following diagnosis, but this did not reach significance levels (HR 0.14, *p* = 0.0505; Additional file 1: Table S3). BRAF Val600Glu mutation status is the only variable identified in this study that was both an early-event (OS and DSS) and late-event (RFS) marker. The reason why this mutation has such effects remains unknown and warrants more investigations. Overall, the wide-spectrum of associations detected for this mutation in this study further strengthen this gene’s importance in colorectal cancer.

#### Adjuvant chemotherapy and radiotherapy treatment status and their relation to outcome measures

Adjuvant therapy is given based on the clinical and disease characteristics to help control the disease (e.g., to reduce/eliminate the recurrence and/or metastasis risk) and to improve the survival outcomes of patients. In our patient cohort, patients who received adjuvant chemotherapy had better survival outcomes (OS, DSS, and EFS) than those patients who did not receive it. These effects were especially stronger within the first year following diagnosis (OS, DSS, and EFS models; Additional file 1: Tables S2-S3, S7). The changing effects (from strong to weaker protective effects) may reflect the slightly diminishing effects of therapy after the treatment duration, which is usually no more than a year [65]. Time-varying effects for chemotherapy treatment were detected in other cancers as well, such as breast cancer [66–68]. On the other hand, adjuvant radiotherapy status was associated with only MFS (Additional file 1: Table S5). Initially, MFS times did not differ significantly for the patients who did or did not receive this treatment. However, after 5.5 years following diagnosis, those patients who received radiotherapy had increased risk of developing their first metastases compared to patients who did not receive this treatment. The exact mechanisms through which adjuvant radiotherapy can have a late effect on MFS of patients is not clear, but it is known that in some cases, radiation treatment increases the risk of metastasis [69–71]. As these authors discussed [69–71], a variety of potential mechanisms can explain this effect, such as the appearance or development of radiation-resistant tumor cells, changes in the tumor microenvironment or immune system response over time, or suppression of the tumor progression by radiation treatment that initially delays the tumor metastasis. These previous and our findings emphasize the need for new research revenues and potentially prolonged surveillance for late-onset metastatic lesions in colorectal cancer patients who are treated with adjuvant radiotherapy.

### Strengths and limitations

Limitations of this study include the missing information on the cause of death for a portion of the patients; assuming that the non-colorectal cancer-related deaths were independent of colorectal cancer; having a small number of recurrences in the dataset, which may have limited the study power in analysis of recurrence-related outcomes; and examining select clinico-demographic and tumor molecular markers, which leaves it to future studies to examine the potential effects of other markers. Additionally, characteristics of the patients who are included in this study may differ from the patients who were diagnosed during the recruitment phase, but declined to consent and participate in NFCCR. This may affect the generalizability of the findings. However, it should also be noted that in some cases, the consent to access the medical records and tissue specimen was obtained from the close relatives/proxies of the patients who had died. Thus, the bias that may be introduced by the exclusion of advanced stage patients is expected to be lower in our study compared to many other studies [35]. This study also has a number of unique advantages: the cohort examined in this study is one of the longest followed up cohorts that allowed the systematic examination of long-term survival characteristics in colorectal cancer; this is a prospective cohort study that reduces information bias compared to retrospective cohort studies [72]; a comprehensive set of outcome measures were examined, which provided detailed information on survival patterns and relationships; and finally, the PH assumption in Cox regression models was checked, and the effects of variables were properly assessed—this not only increased the reliability of the effect estimations, but also allowed us to identify promising early and late effect markers.

## Conclusions

In conclusion, this study describes the long-term survival characteristics of a prospective cohort of colorectal cancer patients and the detailed relationships between baseline variables and patient outcomes over a long time. Overall, our results increase the depth of information on patient outcomes and the markers of short-term and long-term risks and provide new insights that may assist future research and clinical care strategies in colorectal cancer.

## Additional file

Additional file 1: **Figure S1.** Kaplan-Meier curves for the variables with a *p* value < 0.05 in the univariate Cox analyses and with a *p* value < 0.05 in the PH assumption test (type A variables). **Figure S2.** Kaplan-Meier curves for the variables with a *p* value ≥ 0.05 in the univariate Cox analyses and with a *p* value < 0.05 in the PH assumption test (type B variables). **Figure S3.** Kaplan-Meier curves for disease stage. **Table S1.** Pair-wise Pearson correlation coefficient values for the baseline variables. **Table S2.** Associations between clinico-demographic/molecular variables and overall survival (OS) in multivariate analysis. **Table S3.** Associations between clinico-demographic/molecular variables and disease-specific survival (DSS) in multivariate analysis. **Table S4.** Associations between clinico-demographic/molecular variables and recurrence-free survival (RFS) in multivariate analysis. **Table S5.** Associations between clinico-demographic/molecular variables and metastasis-free survival (MFS) in multivariate analysis. **Table S6.** Associations between clinico-demographic/molecular variables and recurrence/metastasis-free survival (RMFS) in multivariate analysis. **Table S7.** Associations between clinico-demographic/molecular variables and event-free survival (EFS) in multivariate analysis. (DOCX 556 kb)

## Data Availability

Data that support the findings of this study are available from Newfoundland Colorectal Cancer Registry/Memorial University but restrictions apply to the availability of this data, and so data are not publically available. The data used in this study cannot be made publicly available as patients were not consented to make their data publicly available or accessible. Data are available from the Newfoundland Colorectal Cancer Registry (NFCCR) for researchers who meet the criteria for access to confidential data. Permission to obtain the data can be requested from Newfoundland Colorectal Cancer Registry (Dr. Patrick Parfrey; pparfrey@mun.ca) and Research, Grant, and Contract Services (rgcs@mun.ca) at Memorial University of Newfoundland, St. John’s, NL, Canada, and the ethics approval shall be obtained from the Health Research Ethics Board (HREB), Ethics Office, Health Research Ethics Authority, Suite 200, 95 Bonaventure Avenue, St. John’s, NL, A1B 2X5, Canada.

## Abbreviations

CI: Confidence interval
DSS: Disease-specific survival
EFS: Event-free survival
HR: Hazard ratio
MFS: Metastasis-free survival
MSI: Microsatellite instability
MSI-H: Microsatellite instability-high
MSI-L: Microsatellite instability-low
MSS: Microsatellite stable
OS: Overall survival
PH: Proportional hazard
RFS: Recurrence-free survival
RMFS: Recurrence/metastasis-free survival
