# Supplementary material for: The long-term survival characteristics of a cohort of colorectal cancer patients and baseline variables associated with survival outcomes with or without time-varying effects
Source: BMC Med. 2019 Jul 29;17:150. doi: 10.1186/s12916-019-1379-5 (PMC6661748; doi:10.1186/s12916-019-1379-5)
Supplement: Supplementary file 1 — Figure S1. Kaplan-Meier curves for the variables with a p value < 0.05 in the univariate Cox analyses and with a p value < 0.05 in the PH assumption test (type A variables). Figure S2. Kaplan-Meier curves for the variables with a p value ≥ 0.05 in the univariate Cox analyses and with a p value < 0.05 in the PH assumption test (type B variables). Figure S3. Kaplan-Meier curves for disease stage. Table S1. Pair-wise Pearson correlation coefficient values for the baseline variables. Table S2. Associations between clinico-demographic/molecular variables and overall survival (OS) in multivariate analysis. Table S3. Associations between clinico-demographic/molecular variables and disease-specific survival (DSS) in multivariate analysis. Table S4. Associations between clinico-demographic/molecular variables and recurrence-free survival (RFS) in multivariate analysis. Table S5. Associations between clinico-demographic/molecular variables and metastasis-free survival (MFS) in multivariate analysis. Table S6. Associations between clinico-demographic/molecular variables and recurrence/metastasis-free survival (RMFS) in multivariate analysis. Table S7. Associations between clinico-demographic/molecular variables and event-free survival (EFS) in multivariate analysis. (DOCX 556 kb) [file 12916_2019_1379_MOESM1_ESM.docx]

**Table S1.** Pair-wise Pearson correlation coefficient values for the baseline variables.

|  | **Sex** | **Histology** | **Location** | **Stage** | **Grade** | **Familial risk** | **MSI status** | **BRAF mutation status** | **Adjuvant chemotherapy** | **Adjuvant radiotherapy** |
| --- | --- | --- | --- | --- | --- | --- | --- | --- | --- | --- |
| **Sex** | 1 | -0.045 | 0.111 | 0.028 | 0.002 | -0.001 | -0.095 | -0.184 | 0.065 | 0.094 |
| **Histology** |  | 1 | -0.069 | 0.103 | 0.11 | 0.030 | 0.082 | 0.107 | 0.038 | -0.062 |
| **Location** |  |  | 1 | -0.082 | -0.018 | -0.028 | -0.175 | -0.231 | 0.235 | 0.663 |
| **Stage** |  |  |  | 1 | 0.179 | 0.038 | -0.117 | 0.037 | 0.127 | 0.003 |
| **Grade** |  |  |  |  | 1 | 0.012 | 0.077 | 0.156 | 0.021 | 0.030 |
| **Familial risk** |  |  |  |  |  | 1 | 0.1 | 0.033 | 0.060 | 0.042 |
| **MSI status** |  |  |  |  |  |  | 1 | 0.368 | -0.011 | -0.085 |
| **BRAF mutation status** |  |  |  |  |  |  |  | 1 | -0.017 | -0.153 |
| **Adjuvant chemotherapy** |  |  |  |  |  |  |  |  | 1 | 0.535 |
| **Adjuvant radiotherapy** |  |  |  |  |  |  |  |  |  | 1 |

MSI: microsatellite instability.

**Fig. S1-S3.** Non-crossing curves of Type A variables (**Fig. S1**) suggest only/mostly protective or detrimental effects but with fluctuating (e.g. increased or decreased) hazard ratios during the follow up while crossing curves of Type B variables (**Fig. S2**) indicate the changed direction of effects (either from protective effect to detrimental effect, or vice versa). These curve patterns are interesting as some of these variables have their effect directions change over time (e.g. BRAF Val600Glu mutation status in DSS), or have their curves clearly separate only during particular time periods (e.g. adjuvant chemotherapy status in EFS) (**Fig. S2**). However, in the absence of an assessment for PH assumption by a proper statistical test, interpretation of Kaplan Meier curve patterns may present themselves as a challenge for the researcher. As **Fig. S2** shows, the crossing nature of the curves may be an initial diagnostics for potential variables, yet for those variables where the curves do not cross (**Fig. S1**), it is more difficult to make an assessment on whether the variable violates the PH assumption. Thus, as also indicated by others (e.g. Quantin et al. 1999), in this study a formal assessment of the violation of the PH assumption in Cox models helped identify the variables with time-varying effects.

Reference:

Quantin C, Abrahamowicz M, Moreau T, Bartlett G, MacKenzie T, Adnane Tazi M, et al. Variation over time of the effects of prognostic factors in a population-based study of colon cancer: comparison of statistical models. Am J Epidemiol. 1999;150(11):1188-200.


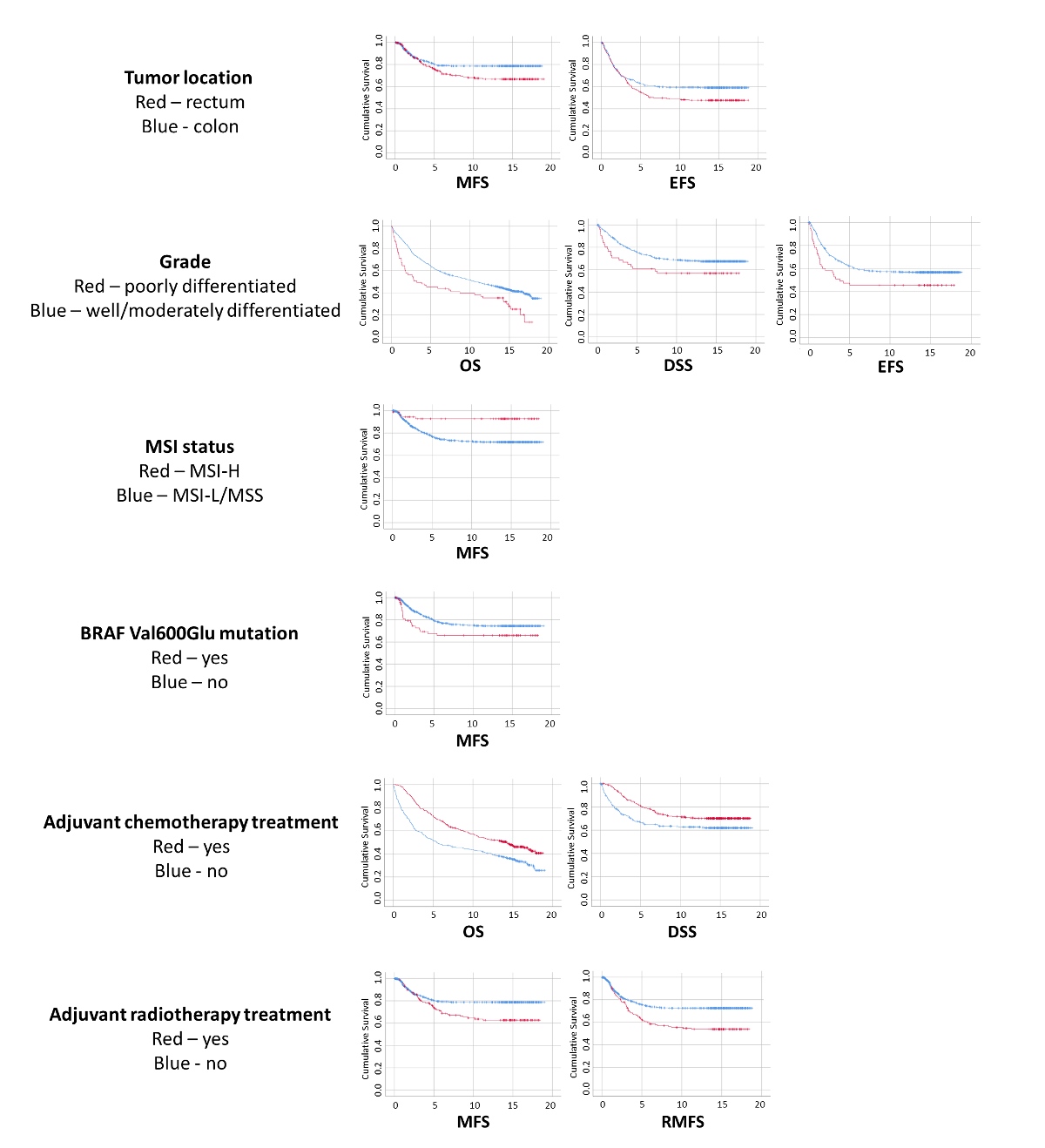


**Fig. S1.** Kaplan Meier curves for the variables with a p-value < 0.05 in the univariate Cox analyses and with a p-value < 0.05 in the PH assumption test (**Type A variables**). DSS, disease-specific survival; EFS, event-free survival; MFS, metastasis-free survival; MSI, microsatellite instability; MSI-H, microsatellite instability high; MSI-L, microsatellite instability low; MSS, microsatellite stable; OS, overall survival; RMFS, recurrence/metastasis-free survival. X-axis shows time in years.


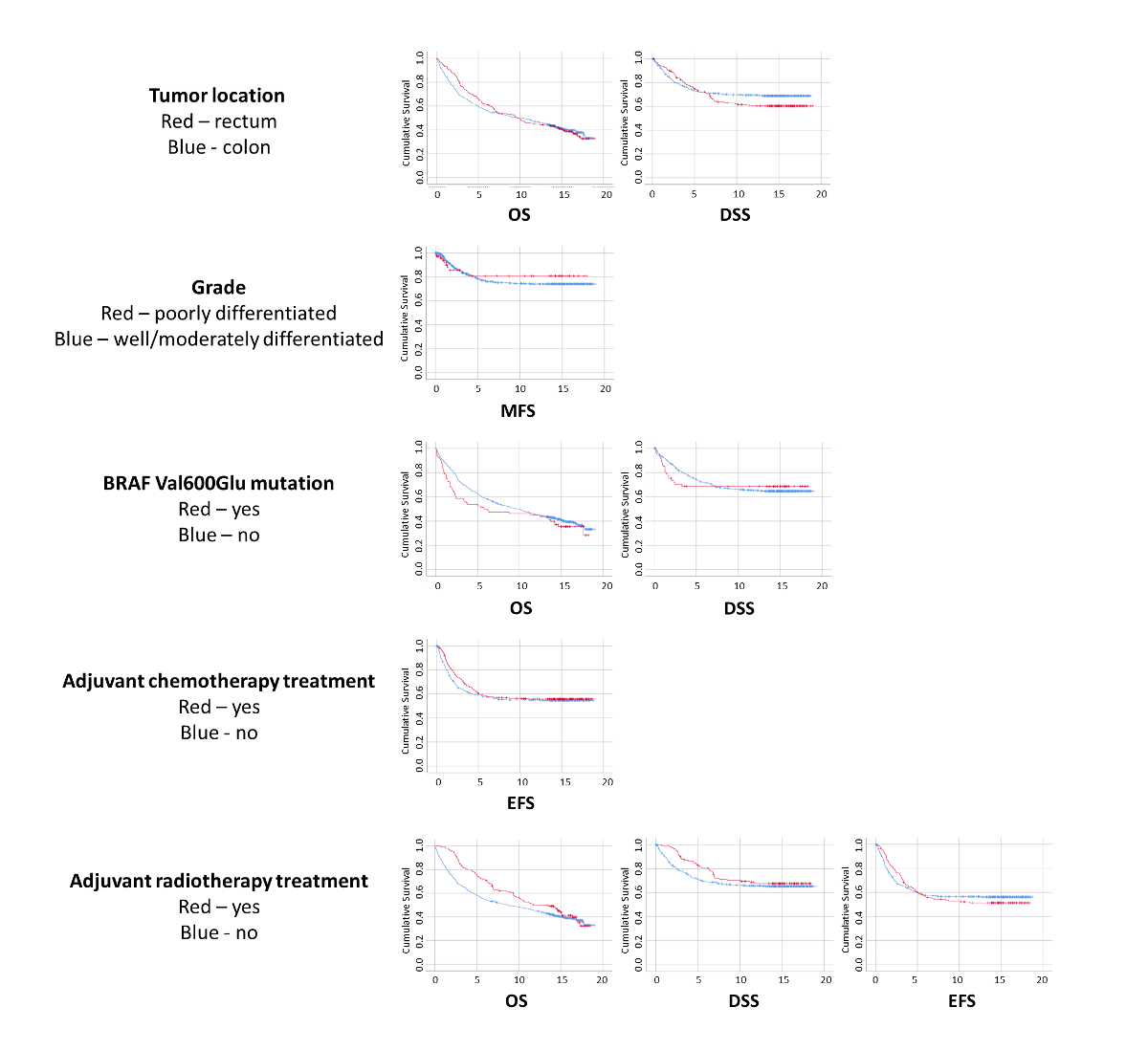


**Fig. S2.** Kaplan-Meier curves for the variables with a p-value ≥ 0.05 in the univariate Cox analyses and with a p-value < 0.05 in the PH assumption test (**Type B variables**). DSS, disease-free survival; EFS, event-free survival; MFS, metastasis-free survival; OS, overall survival. X-axis shows time in years.


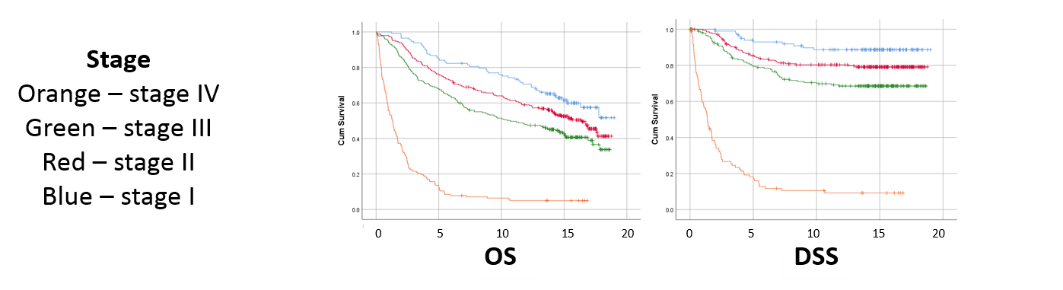


**Fig. S3**. Kaplan-Meier curves for disease stage. The stage III and IV subgroups have p-values < 0.05 in the univariate Cox analysis as well as in the PH assumption tests in the OS analysis, and the stage IV subgroup has the p-value < 0.05 in the univariate Cox analysis as well as in the PH assumption test in the DSS analysis (Type A variable). DSS, disease-specific survival; OS, overall survival. X-axis shows time in years. In this variable, not all variable groups (stage II-IV) violated the PH assumption.

**Table S2.** Associations between clinico-demographic/molecular variables and overall survival (OS) in multivariate analysis.

|  | **Cutoff time point T (year)** | **Time interval** | **HR** | **95% CI for HR (lower)** | **95% CI for HR (upper)** | **p-value** | **p-value for PH assumption test** |
| --- | --- | --- | --- | --- | --- | --- | --- |
| **Age at diagnosis** | 10.5 | Before T | 1.02 | 1.01 | 1.04 | **1.35E-04** | 0.63 |
|  |  | After T | 1.14 | 1.10 | 1.19 | **1.94E-10** | 0.77 |
| **Stage (II vs I)** |  |  | 1.88 | 1.28 | 2.77 | **1.38E-03** | 0.21 |
| **Stage (III vs I)** | 1 | Before T | 38.24 | 12.31 | 118.76 | **2.94E-10** | 0.98 |
|  |  | After T | 3.14 | 1.98 | 4.98 | **1.04E-06** | 0.07 |
| **Stage (IV vs I)** | 1 | Before T | 52.55 | 20.17 | 136.95 | **5.55E-16** | 0.83 |
|  |  | After T | 11.83 | 7.68 | 18.21 | **<2.00E-16** | 0.11 |
| **Location (rectum vs colon)** | 2 | Before T | 0.79 | 0.50 | 1.25 | 0.32 | 0.99 |
|  |  | After T | 1.68 | 1.29 | 2.18 | **1.23E-04** | 0.88 |
| **BRAF Val600Glu mutation status (mutant vs wild-type)** | 2.5 | Before T | 2.18 | 1.47 | 3.23 | **9.54E-05** | 0.65 |
|  |  | After T | 0.70 | 0.43 | 1.13 | 0.15 | 0.70 |
| **Adjuvant chemotherapy treatment (yes vs no)** | 1 | Before T | 0.05 | 0.02 | 0.13 | **1.90E-09** | 0.70 |
|  |  | After T | 0.56 | 0.41 | 0.75 | **1.07E-04** | 0.18 |

CI, confidence interval; HR, hazard ratio; PH, proportional hazard. During model construction for OS, the time cutoff points for stage III and adjuvant chemotherapy were estimated at the same time because a satisfying cutoff time point could not be found for stage III when it was analyzed alone (stage III had the smallest p-value of the PH assumption test on OS and adjuvant chemotherapy had the second smallest one. Testing all the possible combinations of the cutoff time points for these two variables identified one year as the proper cutoff point).

**Table S3.** Associations between clinico-demographic/molecular variables and disease-specific survival (DSS) in multivariate analysis.

|  | **Cutoff time point T (year)** | **Time interval** | **HR** | **95% CI for HR (lower)** | **95% CI for HR (upper)** | **p-value** | **p-value for PH assumption test** |
| --- | --- | --- | --- | --- | --- | --- | --- |
| **Stage (II vs I)** |  |  | 2.51 | 1.25 | 5.03 | **9.46E-03** | 0.33 |
| **Stage (III vs I)** |  |  | 4.99 | 2.34 | 10.62 | **3.06E-05** | 0.13 |
| **Stage (IV vs I)** | 1 | Before T | 85.43 | 25.16 | 290.03 | **9.91E-13** | 0.39 |
|  |  | After T | 21.69 | 10.78 | 43.61 | **<2.00E-16** | 0.14 |
| **Location (rectum vs colon)** | 6.5 | Before T | 1.42 | 0.99 | 2.03 | 0.06 | 0.52 |
|  |  | After T | 5.97 | 2.56 | 13.93 | **3.59E-05** | 0.60 |
| **BRAF Val600Glu mutation status (mutant vs wild-type)** | 2.5 | Before T | 3.05 | 1.79 | 5.19 | **4.09E-05** | 1.00 |
|  |  | After T | 0.14 | 0.02 | 1.00 | * 0.05 | 0.65 |
| **Adjuvant chemotherapy treatment (yes vs no)** | 1 | Before T | 0.15 | 0.04 | 0.50 | **2.28E-03** | 0.80 |
|  |  | After T | 0.50 | 0.33 | 0.77 | **1.79E-03** | 0.32 |

CI, confidence interval; HR, hazard ratio; PH, proportional hazard. The p-values are rounded to two decimals. *The actual p-value = 0.0505.

**Table S4.** Associations between clinico-demographic/molecular variables and recurrence-free survival (RFS) in multivariate analysis.

|  | **Cutoff time point T (year)** | **Time interval** | **HR** | **95% CI for HR (lower)** | **95% CI for HR (upper)** | **p-value** | **p-value for PH assumption test** |
| --- | --- | --- | --- | --- | --- | --- | --- |
| **Location (rectum vs colon)** |  |  | 2.43 | 1.45 | 4.07 | **7.82E-04** | 0.18 |
| **BRAF Val600Glu mutation status (mutant vs wild-type)** | 4 | Before T | 1.33 | 0.51 | 3.48 | 0.57 | 0.66 |
|  |  | After T | 7.10 | 2.52 | 20.00 | **2.04E-04** | 0.75 |

CI, confidence interval; HR, hazard ratio; PH, proportional hazard.

**Table S5.** Associations between clinico-demographic/molecular variables and metastasis-free survival (MFS) in multivariate analysis.

|  | **Cutoff time point T (year)** | **Time interval** | **HR** | **95% CI for HR (lower)** | **95% CI for HR (upper)** | **p-value** | **p-value for PH assumption test** |
| --- | --- | --- | --- | --- | --- | --- | --- |
| **Age at diagnosis** |  |  | 0.98 | 0.96 | 1.00 | * **0.05** | 0.71 |
| **Stage (II vs I)** |  |  | 1.92 | 0.95 | 3.86 | 0.07 | 0.27 |
| **Stage (III vs I)** |  |  | 3.08 | 1.54 | 6.17 | **1.47E-03** | 0.07 |
| **Stage (IV vs I)** |  |  | 1.94 | 0.84 | 4.52 | 0.12 | 0.78 |
| **Location (rectum vs colon)** |  |  | 1.90 | 1.08 | 3.34 | **0.03** | 0.80 |
| **MSI status (MSI-H vs MSI-L/MSS)** |  |  | 0.16 | 0.06 | 0.44 | **4.45E-04** | 0.27 |
| **BRAF Val600Glu mutation status (mutant vs wild-type)** |  |  | 3.46 | 2.06 | 5.82 | **2.77E-06** | 0.11 |
| **Adjuvant radiotherapy treatment (yes vs no)** | 5.5 | Before T | 0.74 | 0.41 | 1.36 | 0.33 | 0.29 |
|  |  | After T | 6.00 | 1.53 | 23.51 | **0.01** | 0.86 |

CI, confidence interval; HR, hazard ratio; MSI, microsatellite instability; MSI-H, microsatellite instability high; MSI-L, microsatellite instability low; MSS, microsatellite stable; PH, proportional hazard. The p-values are rounded to two decimals. *The actual p-value = 0.0496.

**Table S6.** Associations between clinico-demographic/molecular variables and recurrence/metastasis-free survival (RMFS) in multivariate analysis.

|  | **Cutoff time point T (year)** | **Time interval** | **HR** | **95% CI for HR (lower)** | **95% CI for HR (upper)** | **p-value** | **p-value for PH assumption test** |
| --- | --- | --- | --- | --- | --- | --- | --- |
| **Age at diagnosis** |  |  | 0.98 | 0.97 | 1.00 | **0.03** | 0.74 |
| **Stage (II vs I)** |  |  | 2.00 | 1.14 | 3.53 | **0.02** | 0.51 |
| **Stage (III vs I)** |  |  | 3.04 | 1.75 | 5.28 | **7.66E-05** | 0.26 |
| **Stage (IV vs I)** |  |  | 1.76 | 0.84 | 3.69 | 0.14 | 0.99 |
| **Location (rectum vs colon)** | 3 | Before T | 1.49 | 1.00 | 2.23 | * 0.05 | 0.88 |
|  |  | After T | 3.91 | 2.33 | 6.55 | **2.34E-07** | 0.76 |
| **MSI status (MSI-H vs MSI-L/MSS)** |  |  | 0.45 | 0.24 | 0.85 | **0.01** | 0.86 |
| **BRAF Val600Glu mutation status (mutant vs wild-type)** |  |  | 2.87 | 1.81 | 4.56 | **8.12E-06** | 0.63 |

CI, confidence interval; HR, hazard ratio; MSI, microsatellite instability; MSI-H, microsatellite instability high; MSI-L, microsatellite instability low; MSS, microsatellite stable; PH, proportional hazard. The p-values are rounded to two decimals. *The actual p-value = 0.0524.

**Table S7.** Associations between clinico-demographic/molecular variables and event-free survival (EFS) in multivariate analysis.

|  | **Cutoff time point T (year)** | **Time interval** | **HR** | **95% CI for HR (lower)** | **95% CI for HR (upper)** | **p-value** | **p-value for PH assumption test** |
| --- | --- | --- | --- | --- | --- | --- | --- |
| **Stage (II vs I)** |  |  | 2.10 | 1.19 | 3.72 | **0.01** | 0.30 |
| **Stage (III vs I)** | 1.5 | Before T | 6.02 | 2.97 | 12.23 | **6.61E-07** | 0.46 |
|  |  | After T | 2.99 | 1.57 | 5.71 | **9.00E-04** | 0.37 |
| **Stage (IV vs I)** |  |  | 13.18 | 7.56 | 22.96 | **<2.00E-16** | 0.30 |
| **Location (rectum vs colon)** |  |  | 1.80 | 1.36 | 2.37 | **3.10E-05** | 0.11 |
| **MSI status (MSI-H vs MSI-L/MSS)** |  |  | 0.48 | 0.26 | 0.87 | **0.02** | 0.67 |
| **BRAF Val600Glu mutation status (mutant vs wild-type)** |  |  | 2.21 | 1.49 | 3.26 | **7.13E-05** | 0.52 |
| **Adjuvant chemotherapy treatment (yes vs no)** | 1 | Before T | 0.40 | 0.22 | 0.72 | **2.15E-03** | 0.82 |
|  |  | After T | 0.88 | 0.61 | 1.26 | 0.48 | 0.94 |

CI, confidence interval; HR, hazard ratio; MSI, microsatellite instability; MSI-H, microsatellite instability high; MSI-L, microsatellite instability low; MSS, microsatellite stable; PH, proportional hazard.
